# Coronary Artery Aneurysms: A Clinical Case Report and Literature Review Supporting Therapeutic Choices

**DOI:** 10.3390/jcm13185348

**Published:** 2024-09-10

**Authors:** Michele Sannino, Matteo Nicolai, Fabio Infusino, Luciani Giulio, Tommaso Leo Usai, Giovanni Biscotti, Alessandro Azzarri, Marina De Angelis D’Ossat, Sergio Calcagno, Simone Calcagno

**Affiliations:** 1 Cardiology Unit, Department of Emergency and Admission, San Paolo Hospital, Largo Donatori di Sangue 1, 00053 Civitavecchia, Italy; michele.sannino@uniroma1.it (M.S.); giulio.luciani@aslroma4.it (L.G.); tommaso.usai@aslroma4.it (T.L.U.); giovanni.biscotti@aslroma4.it (G.B.); alessandro.azzarri@aslroma4.it (A.A.); sergio.calcagno@aslroma4.it (S.C.); 2 Radiodiagnostic Unit, San Paolo Hospital, Largo Donatori di Sangue 1, 00053 Civitavecchia, Italy; matteo.nicolai@aslroma4.it (M.N.); marina.deangelisdossat@aslroma4.it (M.D.A.D.); 3 Division of Cardiology, S. Giovanni Evangelista Hospital, 00019 Tivoli, Italy; fabio.infu@gmail.com

**Keywords:** coronary abnormalities, dilatation, thrombosis, coronary disease, atherosclerosis

## Abstract

Coronary artery aneurysms (CAAs) are uncommon but significant cardiovascular abnormalities characterized by an abnormal increase in vascular diameter. CAAs are classified based on their shape as either saccular or fusiform, and their causes can range from atherosclerosis, Kawasaki disease, to congenital and iatrogenic factors. CAAs often present asymptomatically, but when symptoms occur, they can include angina, myocardial infarction, or even sudden cardiac death due to intravascular thrombosis involving the CAA. Diagnosis is typically confirmed through coronary angiography, though CT and other imaging techniques can provide additional details. The management of CAAs is variable depending on their size, location, and the presence of symptoms or complications. Treatment options include medical therapy, percutaneous coronary intervention (PCI), or surgical approaches. In this paper, we describe the case report of a 79-year-old male who presented with palpitations and was diagnosed with a right coronary artery aneurysm, and a review of the literature is delineated, underscoring the importance of individualized treatment strategies for CAAs.

## 1. Introduction

A coronary artery aneurysm is a rare finding in catheterization laboratories during coronary angiography. Despite the poor data, a specific definition and classification were advanced by heart and cardiovascular associations. CAAs are typically described when the enlargement extent of coronary sections is more than or equal to 1.5 times that of the closest normal arterial portion. More rarely, the vessel diameter reaches 20 mm, 40 mm, or 50 mm, or four times the reference vessel, and is defined as a giant CAA (GCAA) [1,2]. The etiology of CAAs is still unclear. Atherosclerosis is the most frequent risk factor in adults, but the larger incidence of these inappropriate vessel dilatations is found in the pediatric population affected by Kawasaki disease and Takayasu arteritis. More frequently, the presence of a CAA is found during coronary CT or coronary angiogram, and it is not related to a specific coronary syndrome. Occasionally, a CAA can become complicated, developing intravascular thrombosis, distal embolization, vessel rupture with a consequent myocardial ischemia, heart failure, and arrhythmias. The management of CAAs remains a challenge for interventional cardiologists due to the absence of clear guideline indications. The choice of optimal medical management, percutaneous coronary intervention, or surgical procedure is still not defined. Therefore, this literature review purposes to unite the current information on CAAs, aiming to increase the knowledge and reach a clearer line of treatment.

### 1.1. Definition and Classification of Coronary Artery Aneurysms

Coronary artery aneurysms (CAAs) are usually defined when the dilation extent of coronary segments is more than or equal to 1.5 times that of the adjacent normal arterial segment [3,4]. Despite the first CAA being found by Morgagni in 1761, current knowledge is still limited. Therefore, a clear treatment line is not defined. Coronary vessel dilatation is occasionally so large that it called a giant CAA (GCAA). The diameter values used to classify GCAAs are different in adult and young patients. In adults, the diameter of the vessel must reach a value of >4 times or >20 mm, whereas in child patients an enlargement >8 mm of the coronary lumen is considered a GCAA. In the patient setting of the latter group, the proposed sizes were advanced by the American Heart Association and are mostly applied on Kawasaki disease, which is the more frequent cause of GCAAs in children [5].

Another classification is based on the shape of the CAA, and two different types have been proposed: a saccular aneurysm and fusiform aneurysm. The first has a spherical-shaped distension, characterized by a transverse diameter > longitudinal diameter; in the second type, the longitudinal diameter is larger than the transverse diameter, describing a spindle shape [6].

CAAs have an incidence ranging from 0.35% to 5% in the general population, and their diagnosis can be made in both elective patients, as incidental findings, and in those with acute coronary syndrome (ACS) during an emergency procedure [7]. In recent years, the large use of multi-slice computed tomography (MSCT) on coronary arteries has led to an increase in the frequency of CAAs found in patients undergoing coronary imaging, whether invasive or not.

There is a gender difference in the incidence of CAAs, with a prevalence in men rather than in women [7].

CAAs are more frequent in the right coronary artery, while they are rarer in the left main coronary artery, with a rate of only 0.1%. The coronary portions more often affected are the proximal or mid segment, rather than the distal segment of the vessels [8].

### 1.2. Risk Factors

The risk factors for developing a CAA are atherosclerosis, inflammation, and non-inflammation causes. Atherosclerosis is the most frequent risk factors in adults, reaching a frequency of 50% in all CAAs, independent of the presence of coronary artery plaque [9]. Kawasaki disease (KD) is the most common cause of inflammatory CAAs in child patients and the second most frequent cause in adult patients [7].

CAAs can present in other syndromes, such as connective tissue disease (Marfan syndrome, Ehlers-Danlos syndrome, fibromuscular dysplasia, neurofibromatosis), vasculitis (lupus, rheumatoid arthritis, ankylosing spondylitis, scleroderma), infections (human immunodeficiency virus, bacterial, mycobacterial, syphilis, Lyme disease, mycotic aneurysm, septic emboli), drug use (cocaine, amphetamine, protease inhibitors), neoplastic lesions, and cardiac lymphoma.

Despite the real pathophysiological mechanism remaining unclear, a possible explanation for atherosclerosis developing CAAs could be related to the reduced resistance to intravascular stress, causing a progressive enlargement of a specific tract of the artery where the share-stress is more persistent. Not all patients with atherosclerosis have CAAs, so a genetic predisposition is probably needed. A previous study identified a possible role of a specific allele of the MMP gene—MMP3-5A, codifying for enzymes involved in the proteolysis of connective tissue proteins—because it was more prevalent in patients with CAAs [10]. Other causes may be congenital (fibromuscular dysplasia), iatrogenic, drug-related, or infective.

Adding to these conventional risk factors, 0.2–2.3% of patients who have undergone PCI described the development of CAAs after this procedure [11]. CAA growth seems to be related to drug-eluting stent implantation, long lesion, and anterior intraventricular artery stenting [12]. In particular, several cases have been reported following the use of stent or drug eluting balloon (DEB), both with the drugs sirolimus and everolimus [12,13]. The development of an aneurysm after DEB treatment might have different causes from CAA formation after DES implantation. The combination of an elevated concentration of a drug that strongly induces cell apoptosis and the mechanical trauma on the endothelium may be the key reason for aneurysm formation [14]. Moreover, other mechanisms seem to be involved in regional positive vascular remodeling, such as the high drug concentration and its rapid local release on the vessel wall, thus worsening the incomplete therapeutic and inflammatory processes secondary to the negative effects of this device.

More recently, aneurysm formations after BVS implantation have been documented in case reports and retrospective registries [15,16,17,18,19] and have been observed up to 32 months after BVS implantation [20]. The real incidence of aneurysm formation after BVS implantation remains unknown. The pathophysiology and evolution of artery enlargement after BVS implantation are not fully understood. The same factors have been reported as a possible mechanism of coronary evagination or aneurysm dilatation after BVS deployment: dissections and deep vessel injury, inflammation, and allergic reaction stimulated by the drug, the polymer, or the device itself, pathological vessel healing with excessive remodeling, and even focal infections likely to contribute to aneurysm formation [21]. Regarding the aneurysms, a study reports that the correlation between BVS and the development of coronary evagination has an incidence comparable to that of contemporary DES [15]. Gori et al. observed evaginations in 54% of BVS cases, and aneurysms in three patients; an imaging analysis with OCT showed a strong association with BVS malposition and strut fractures [18]

### 1.3. Clinical Presentation

The presence of CAAs is mostly asymptomatic. Indeed, the symptoms depend on cause-related factors: size, compression, concomitant artery stenosis, rupture, thrombosis, distal embolization, or fistula formation [11,21,22]. Angina is the most recurrently experienced symptom in patients with CAAs, and in rarer cases dyspnea can occur. The clinical presentation is highly variable, including stable and unstable angina, non-ST or ST-elevation myocardial infarction (NSTEMI or STEMI), alteration in rhythm, sudden cardiac arrest, or congestive heart failure [21]. Angina symptoms are not exclusively related to the presence of coronary artery plaques but are associated with compromised flow into the aneurysm with a reduction in myocardial perfusion sometimes due to exercise and stress [23].

In most cases, CAAs remain asymptomatic and unchanged by size and dimension. In rare cases, patients can develop acute coronary syndrome (ACS) due to a possible complication of the aneurysm. The alteration in blood flow into a CAA can result in thrombus formation, especially if the diameter is >5 mm. Distal embolization is the more frequent cause of ACS development [24], highlighting a direct correlation between large aneurysm size and an increased risk of developing intraluminal thrombosis [24].

Despite this, the data on ACS are ambiguous, and worsening prognosis is directly associated with coronary artery obstructive disease extension. In respect to CAAR, authors have shown that the mortality rate is 15.3% and the incidence of major adverse cardiac events (MACEs) is 31%, especially if CAAs are associated with severe and diffuse coronary artery stenosis [25].

In rarer cases, the worst developments consist of dissection, coronary break, ACS due to large thrombus burden, and pericardial effusion with cardiac tamponade. In the case of giant CAAs, the compression of surrounding structures can occur.

### 1.4. Diagnostic Skills

Both invasive and non-invasive vascular imaging may be used as diagnostic tools. Coronary angiography (CAG) is the gold standard procedure. It can give information about shape, size, and possible complications such as blood flow alterations, fistula development, and external mass compression. Moreover, possible treatments can be performed during the same procedure, excluding aneurysm with PCI.

CAG cannot provide information about the wall vessels, underestimating their size, and in some circumstances CAAs may be neglected in cases of occlusion or the presence of intraluminal thrombus [26]. In other situations, intracoronary imaging by IVUS may be very helpful and can provide information, such as on the presence or absence of all wall layers and luminal composition, in order to detect endoluminal thrombosis [27]. Underlining the importance of intracoronary imaging, in a recent study, Maehara and coauthors [28] performed an IVUS scan in 77 consecutive patients with aneurysmal dilatation in a native coronary artery in order to clarify the morphology. The data showed that about 30% of the analyzed aneurysms were real aneurysms or pseudoaneurysms, and the other lesions had the morphology of complex plaques with adjacent stenosis.

Another invasive imaging technique is optical coherence tomography (OCT), but its use may be reduced in evaluating CAAs due to its lower scanning diameter and large quantity of contrast medium needed to obtain a significant scan [28].

Regarding not-invasive techniques, computed tomography (CT) is the most useful one because it can provide information about size, morphology, and location [29]. Furthermore, 3-dimensional reconstructions simplify the interpretation of the connection between the aneurysm and contiguous structures [29]. CT may be used in follow-up assessments due to the presence of possible complications and the ability to perform it in outpatient clinics.

Of the other non-invasive imaging techniques, cardiac magnetic resonance (CMR) imaging is an important method for identifying CAAs, as an alternative to coronary CT. In addition to being non-invasive and not requiring ionizing radiation or iodinated contrast agents, coronary artery CMR provides a definitive three-dimensional (3D) “road map” of the mediastinum. Moreover, coronary artery CMR is often preferred for follow-up because there is no need for ionizing radiation or intravenous contrast, especially in young patients. Echocardiography can also be used, but, considering the well-known limits of echocardiography (operator-dependent, chest patient’s echogenicity, and imprecise evaluation of mid and distal sections of coronary arteries), this technique is not fully diffuse. The echocardiography criteria are not standardized, but some findings can help with diagnosis, especially in giant CAAs, mainly if it is performed in three dimensions. During echocardiography evaluation, finding a saccular or fusiform ring with an echo-free core, smooth cystic mobile mass, and an alteration in velocity blood flow by color Doppler in different cutting planes is helpful in the diagnosis of vascular structures. In addition, examination when agitated saline contrast medium is injected excludes the presence of intracardiac structures. In specific situations, such as with Kawasaki disease or the other syndromes mentioned above, echocardiography can identify CAAs in the proximal section of the left main coronary artery and the right coronary artery [30].

Congenital and genetic CAA correlations should be found in young patients, in patients with specific syndromes, and in those with a familiarity of autoimmune and vasculitis diseases. Vasculitis presents several diagnostic challenges. Indeed, patients can present with variable clinical manifestations and an extensive spectrum ranging from isolated cutaneous vasculitis to multisystem involvement. Moreover, there are numerous medical disorders that can mimic vasculitis, like common infections. Therefore, vasculitis in patients with a diagnosis of CAA should be supposed in patients developing symptoms or signs of myocardial ischemia, especially if cardiovascular risk factors and atherosclerotic evidence are missing. Moreover, vasculitis should be detected in young patients with prolonged fever associated with non-infective conditions or those unresponsive to antibiotic therapy. Since the therapy of some vasculitis cases is specific, it is important to rapidly proceed to a differential diagnosis. Diagnosis advances with laboratory and cultural blood and urine tests. In specific situations, biopsy in search of specific findings is useful to identify specific types of vasculitis. But because a pathognomonic test still does not exist, diagnosis continues to rest on the identification of the main clinical outcomes and the exclusion of other clinically analogous entities with recognized causes.

### 1.5. Treatment

Since there is no clear indication in the literature for the management of coronary artery aneurysm, an approach based on individual clinical cases is reasonable. Possible management options include medical therapy and follow-up, percutaneous interventional procedures, or surgical approaches.

A conservative approach is considered a feasible choice in asymptomatic patients without other indications for cardiac surgery or indications related to the aneurysm (i.e., fistula formation, compression, giant aneurysms, left main and saphenous vein graft involvement, rapid size increase, or Kawasaki disease) or if percutaneous intervention or surgical approaches are not feasible. Even in these cases, medical therapy is not unequivocally described because of the absence of a strong indication in the literature. Given the understanding of the pathophysiology of coronary aneurysms and evidence of an increased expression of endothelial integrins with pro-aggregating effects on platelets, antiplatelet therapy is widely used.

Acetylsalicylic acid, administered once daily at a dose of 100 mg, is the most widely used therapy in the literature for both adults and the pediatric population. Clopidogrel, administered once daily at a dose of 75 mg, is chosen as the first-line therapy in about one-third of patients without an indication for percutaneous or surgical treatment. The use of antiplatelet therapy compared to anticoagulation is supported by data from the Coronary Artery Aneurysm Registry (CAAR study), which conducted an average follow-up of 37.2 months in approximately 1500 patients [25]. In this study, antiplatelet therapy was the most used drug, and aspirin was the most prescribed treatment (90.2%) alone or in association with another antiplatelet agent in 64.8% of patients for 12 months. In 13.4% of patients with CAAs, an increased risk of thrombosis was needed to add an anticoagulation drug or an extended dual antiplatelet therapy (DAPT) [25]. These strategies showed a lower mortality rate in a previous study in patients treated with DAPT or antithrombotic drugs [5,31,32].

Additionally, due to the presence of blood stasis at the sites of aneurysmal lesions frequently associated with slow flow in the distal portion of the vessel involved, several authors propose the use of anticoagulant drugs to avoid thrombosis formation within the aneurysmal sac. Anticoagulant therapy is indicated for patients with Kawasaki disease, and the only treatments with an indication are vitamin K antagonists. Despite the absence of data, it is reasonable to believe that in patients who have another indication for taking DOACs, the combination with antiplatelet therapy should be reserved for extremely select cases with a low risk of hemorrhage and an elevated thrombotic risk, in order to decrease the risk of major bleeding events [31]. In a specific subanalysis of the CAAR registry, comparing the use of antithrombotic and antiplatelet therapy failed to show a superiority of one strategy vs. the other. The incidence of major adverse cardiac events (MACEs) was 39.0% in the oral anticoagulation therapy (OAT) group vs. 35.9% in the non-OAT group (*p* = 0.47); further, the rates of net adverse clinical events (NACEs) were similar the among groups (44.6% in the OAT group vs. 39.2% in the non-OAT, *p* = 0.21) [32].

Despite the analysis of all these data, the optimal antithrombotic strategy is not still clear. The use of antiplatelet or anticoagulation drugs, in the absence of other indications for OAT, is not perfectly delineated. We can only conclude that in the presence of large aneurysms associated with slow flow or the slow washout of contrast medium, or evident thrombotic material inside the aneurysm, the prescription of OAT seems to be safer than antiplatelet therapy. This indication is not expressed by guidelines or consensus/opinion leader, and it can only be deduced by interpretation of the data reported above. On the contrary, if the thrombosis is treated with DES or coverage stent, the therapy requires DAPT. This underlines that an optimal medical therapy for all patients is not definitive, and that only in specific situations and particular anatomic conditions can we prefer one therapy or the other, as expressed above.

PCI and coronary angioplasty are now the most widely used techniques for the management of coronary atheromatous disease, but despite their widespread use, there are still limited data on the treatment of CAAs, especially in symptomatic patients. Some studies have found a lack of statistically significant differences between patients treated with angioplasty and those treated with coronary artery bypass grafting [23]. Regarding patients who experience an infarction related to the presence of an aneurysm, the data report an increase in major cardiovascular events compared to patients with infarction without an aneurysm. This can easily be explained by the greater difficulty of treatment due to altered coronary anatomy. Given the pathophysiology of infarction related to aneurysm, the primary goal is to reduce the amount of thrombotic material using thromboaspiration devices, although in cases where it is not possible to restore flow, the implantation of covered stents in the ectasis segment may be useful. The CAAR study showed a significant increase in MACEs in patients in whom bare metal stent (BMS) was used; therefore, these types of devices are reasonably discouraged. In the case of saccular aneurysms, coil embolization or covered stents may be useful if a collateral branch is not involved in the lesion. In asymptomatic patients or those with stable angina, there are still insufficient data to suggest a first-choice treatment among PCI, CABG, or medical therapy [33,34,35].

Moreover, aneurysm and other coronary stenoses can be treated by either a PTFE-covered stent, alongside other drug-eluting stent implantations in tracts outside the aneurysm, or the so-called ‘stent-assisted’ embolization, which involves percutaneous treatment using membrane-covered stents and coil embolization, with a strong limitation in patients with large or multiple CAAs [36]. Another described off-label technique is the use of carotid stent implantation for the treatment of CAAs, but very few data exist and are reported in the literature [37].

There are few indications that make cardiac surgery the first-choice therapy, specifically including other cardiac surgical indications (valvular diseases or critical atheromatous disease requiring CABG); aneurysms involving large collateral branches; left main aneurysm; giant or multiple aneurysms; mechanical complications, compression of structures, or fistula formation; signs of rupture; Kawasaki disease or infected aneurysms; symptomatic venous graft aneurysms; or those causing significant downstream flow reduction [5,9]. Of course, surgery is the only option in patients where the percutaneous approach is technically risky or excessively complex. Surgical treatment consists of aneurysm resection, marsupialization with interposition graft, aneurysm asportation, or thrombectomy associated with CABG [1,38]. The choice between PCI and surgical strategies is often challenging, and it is driven by many factors, such as diffuse coronary artery disease resulting in multivessel disease, left main involvement, or diabetes. Moreover, surgical therapy is preferred in cases of mechanical complications, such as fistula, compression, or rupture. In any case, the optimal treatment strategy, among those proposed above, should be discussed by a heart team and shared with the patient and his/her family.

**Figure 1:**
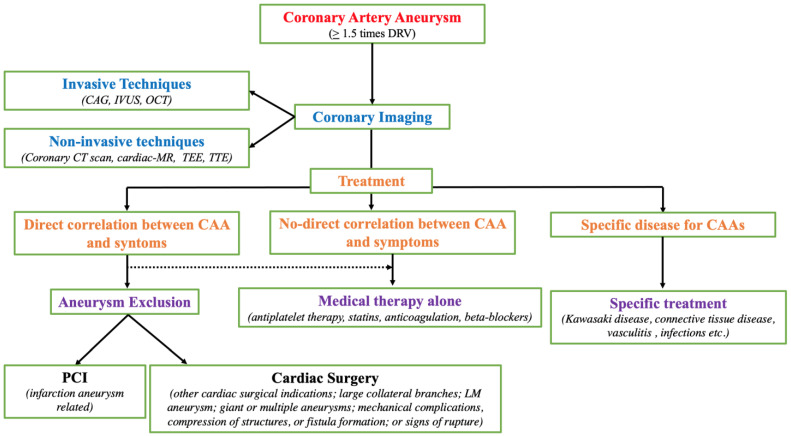
Flowchart for the diagnosis and treatment of coronary artery aneurysm (CAA). DRV diameter of the reference vessel; CAG: coronary angiography; IVUS intravascular ultrasound; OCT optical coherence tomography; MR magnetic resonance; TEE trans-esophageal echocardiography; TTE: transthoracic echocardiography; PCI percutaneous coronary intervention; LM left main.

We propose an algorithm for coronary artery aneurysm (CAA) management to underline the importance of coronary imaging in completing diagnostic assessment and choosing the correct treatment approach (Figure 1).

### 1.6. CAAs in Kawasaki Disease

Kawasaki disease (KD) is an acute, febrile illness of unknown origin and it is the most common cause of acquired heart disease in children in developed countries [5]. Long-term prognosis is determined by coronary artery involvement, but timely initiation of treatment has reduced the incidence of coronary artery aneurysms from 25% to 4%. The first incidence of Kawasaki disease was described in Japan, but its importance concerns the whole world, with an annual incidence of 243.1 per 100,000 children in Japan and 25 per 100,000 children in the continental United States. The KD has a fatality rate of 0.015% in Japan, with peak mortality occurring 15 to 45 days after the onset of fever [39].

Despite the fact that certain causes of KD are still unknown, a possible trigger could be identified in the inflammatory and immune response to any antigen, and RNA viruses of the respiratory system seem to be principally responsible [40,41,42]. Moreover, the genetic intersegment of KD development has been advanced, considering the increased incidence among Japanese children, children of Japanese descent residing outside of Japan, and the increased incidence in family members of an index case. Genomic analyses have been performed, showing that specific alleles and mutations correlate with an increased risk of KD.

KD has systemic involvement, but vasculopathy is the most important and common presentation. Large or giant coronary artery aneurysms frequently accompany vascular alteration in these patients. Cardiovascular manifestations can be conspicuous during the acute KD phase and are the principal cause of long-term morbidity and mortality. All cardiac structures can be involved, including the pericardium, myocardium, endocardium, and coronary arteries. Structural valvular disease occurs in a quarter of patients with an increased involvement of the mitral valve [43]. Coronary artery anomalies are considered a specific diagnostic criterion, principally for patients without clinical criteria. These vessel alterations, including diameter dilation alone or vascular aneurysms, start from the proximal tracts of coronary arteries and then reach the distal segments. Anatomic resolution appears within 4 to 8 weeks in the majority, 32–50%, of patients due to the resolution of inflammation or the causes connected to the fever and circulating inflammatory mediators [44,45].

Clinical manifestations are also infrequent in patients with diffuse coronary disease. The symptoms appear with myocardial ischemia secondary to coronary artery flow alteration or thromboses, or following complications such as the rupture of a coronary artery aneurysm.

Treatment includes a specific therapy, with intravenous immunoglobulin (IVIG) administered in the acute phase of KD and other additional drugs (described above) used to prevent the complications associated with cardiac involvements and symptoms.

## 2. Clinical Case Presentation

A 79-year-old male patient was admitted to our emergency department for arrhythmic palpitations associated with lipothymia, without a complete loss of consciousness. An electrocardiogram revealed atrial fibrillation with a mean ventricular rate of 125 bpm, left axis deviation, and diffuse and nonspecific ventricular repolarization abnormalities. Arterial blood gas analysis showed mild hypoxemia, normocapnia, normal lactate levels, and a slight reduction in circulating bicarbonates. Routine blood tests revealed normal creatinine levels, mild anemia, and elevated levels of high-sensitivity troponin, despite their stability in two determinations (180→190 ng/L) without an ascending and/or descending curve trend (normal value < 14 ng/L).

Given the findings of myocardial injury according to the Fourth Universal Definition of Myocardial Infarction and the presence of undated atrial fibrillation, the patient was transferred to the Cardiac Intensive Care Unit of our department.

### 2.1. Medical History

The patient had systemic arterial hypertension, dyslipidemia with a correct control of LDL levels, and he was a current smoker. A moderate bilateral carotid atheromasia with 50% stenosis in the right common carotid artery was reported. No previous cardiovascular events were reported. Considering the diagnosis of atrial fibrillation with CHA2DS2-VASc = 4, anticoagulation therapy was mandatory to prevent any cerebral embolic events, and therapy with a direct oral anticoagulant (DOAC) drug was started.

### 2.2. Examinations

During hospitalization, a transthoracic echocardiogram (TTE) was performed, revealing mild concentric left ventricular hypertrophy and normal systolic function without wall-motion abnormalities (EF 55%).

**Figure 2:**
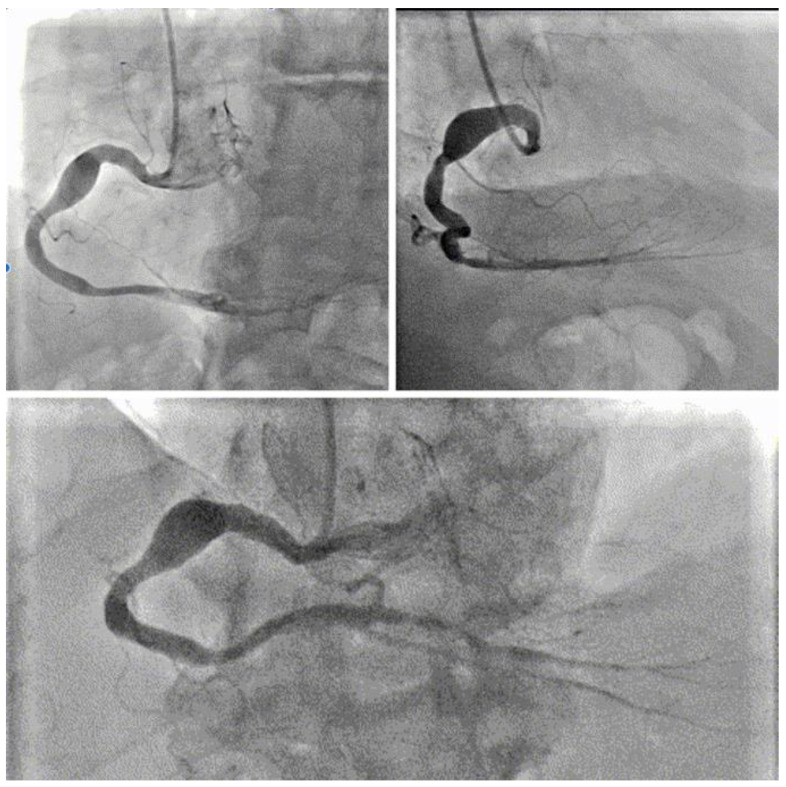
Coronary angiography in three different projections of the right coronary artery (in order: LAO 30, RAO 30, LAO 30 CRAN 30) showing an aneurysmal formation in the mid–proximal segment.

Given the clinical picture, in order to exclude an ischemic nature to the myocardial necrosis marker alteration, a coronary angiography was performed, which showed a normal origin of the coronary arteries, right dominance of coronary circulation, and the presence of an aneurysm of the right coronary artery at the level of the proximal segment, with a diameter of approximately 9 mm and a length of 20 mm, without angiographically significant stenosis (Figure 2). More specifically, the aneurysm started from the proximal segment and reached the middle segment of the right coronary artery. There was slow contrast medium runoff at the level of the aneurysmal portion but no signs of slow flow distal to the lesion (TIMI III), and we did not find any signs of intravascular thrombosis in the aneurysm lumen. The left coronary artery did not show significant stenosis. Due to the absence of chest pain, wall motion abnormalities, significant flow alterations downstream of the aneurysm, and rupture signs, we decided not to perform stent placement at the level of the aneurysm.

### 2.3. Management

**Figure 3:**
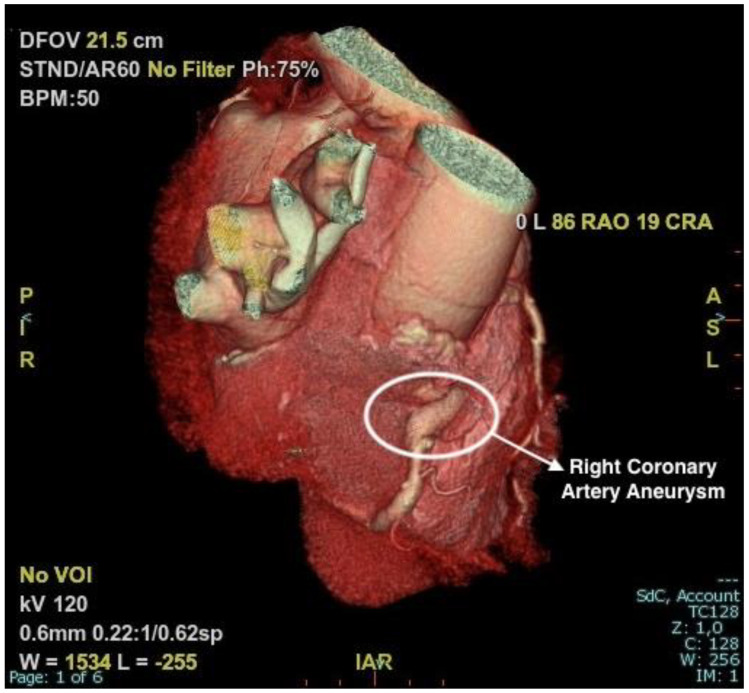
Three-dimensional reconstruction of CT for viewing the right coronary artery, highlighting the aneurysmal formation in the middle segment and its relationship with surrounding anatomical structures.

**Figure 4:**
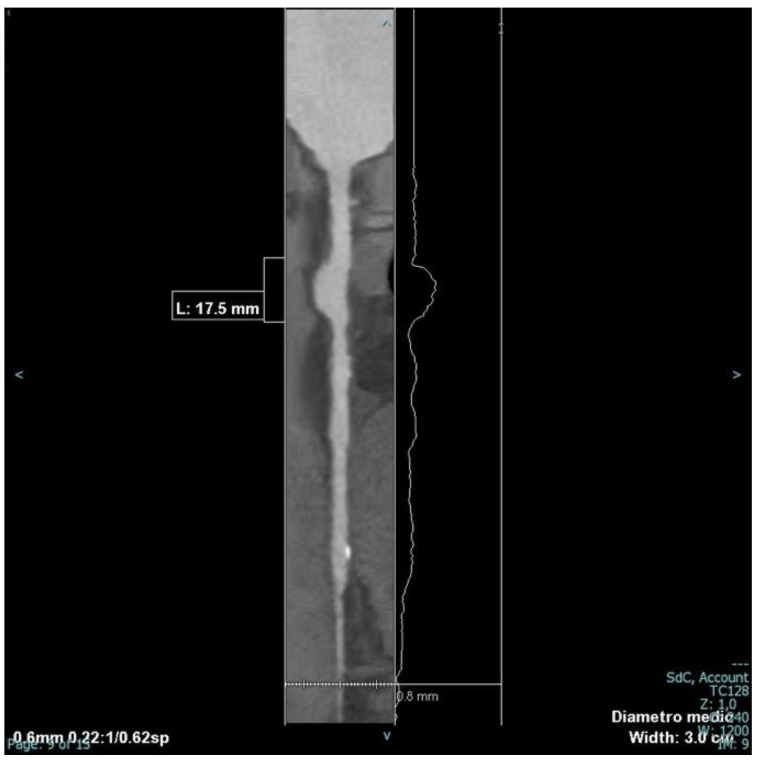
Two-dimensional reconstruction of the right coronary artery to allow for a more accurate measurement of the length and maximum diameter of the aneurysmal segment.

Given the absence of thrombosis and clear indications in the literature regarding the treatment of coronary aneurysms, the team decided to perform a coronary angio CT for a baseline assessment, complete the diagnosis procedure, obtain the correct dimensions of the coronary aneurysm, and check if there were any signs of rupture or thrombosis of the aneurysmal sac. The CT scan confirmed the presence of an aneurysmal formation at the proximal segment of the right coronary artery, with a maximum diameter of 9 mm and a length of approximately 18 mm (Figure 3 and Figure 4). No signs of rupture or thrombosis of the aneurysmal sac were evident with this technique.

In the case described, the patient had never experienced angina, and the increase in myocardial necrosis markers occurred concurrently with elevated blood pressure values and atrial fibrillation. Additionally, there were no signs of thrombosis and/or distal embolization, and slow contrast medium flow was present in all coronary arteries, not just the right coronary artery, which pointed to other reasons of slow washout being more likely, such as microcirculatory dysfunction or high pressures in the coronary venous system. Evaluated with coronary angio CT, the absence of wall thickness loss in the aneurysmal portion of the vessel seemed to justify a conservative follow-up approach in this case.

It was decided to perform clinical and instrumental follow-ups with transthoracic echocardiogram and coronary angio CT. Antihypertensive therapy was intensified in order to maintain systolic blood pressure in the range of 120–130 mmHg and diastolic blood pressure in the range of 70–80 mmHg. The presence of atrial fibrillation with a CHA2DS2-VASc score of 4 indicated the use of direct oral anticoagulants (DOACs) in class I evidence A. Therefore, therapy with apixaban (5 mg bis in die) was initiated without introducing an antiplatelet agent to minimize the risk of bleeding events.

## 3. Conclusions

CAAs are rare and occasional findings, and atherosclerosis presents the principal cause of CAAs, except for in cases with specific syndromes. Congenital and genetic CAA correlation should be sought in young patients, in patients with specific syndromes, and in those with familiarity to vascular and autoimmune diseases. Despite the complications related to CAAs, they are rare, and the potential risk of developing MACEs or cardiac death is mainly higher in patients with diffuse coronary artery disease. Considering the literature data on the rate of events, coronary artery aneurysms can be classified as a specific set of coronary artery diseases. Given the lack of definitive indications for the treatment of coronary ectasias and coronary aneurysms, we believe that an approach based on individual clinical cases and anatomic characteristics is appropriate. Medical therapy and follow-up are valid options and should be chosen as the first interventions in asymptomatic patients in whom lesions are incidentally diagnosed. PCI or cardiac surgery are alternatives in specific situations, especially if the CAA presents signs of possible complications or clinical impairment.
